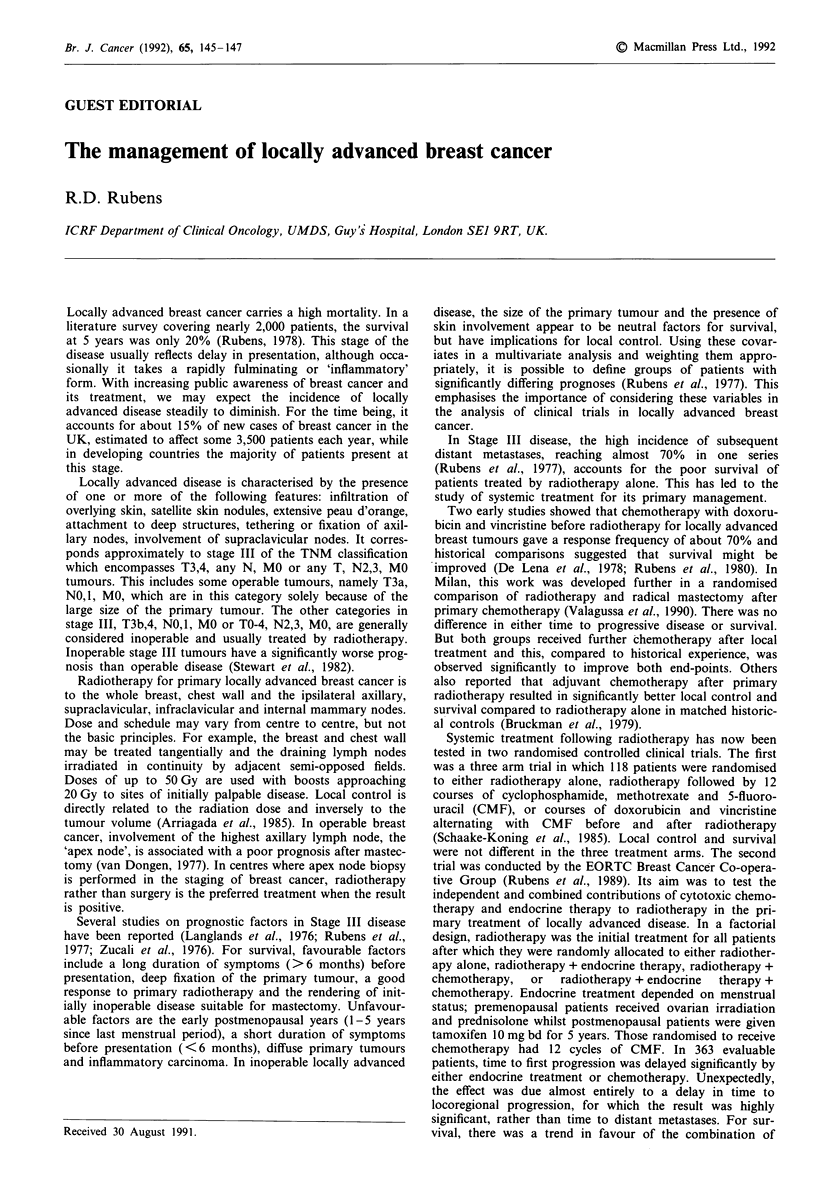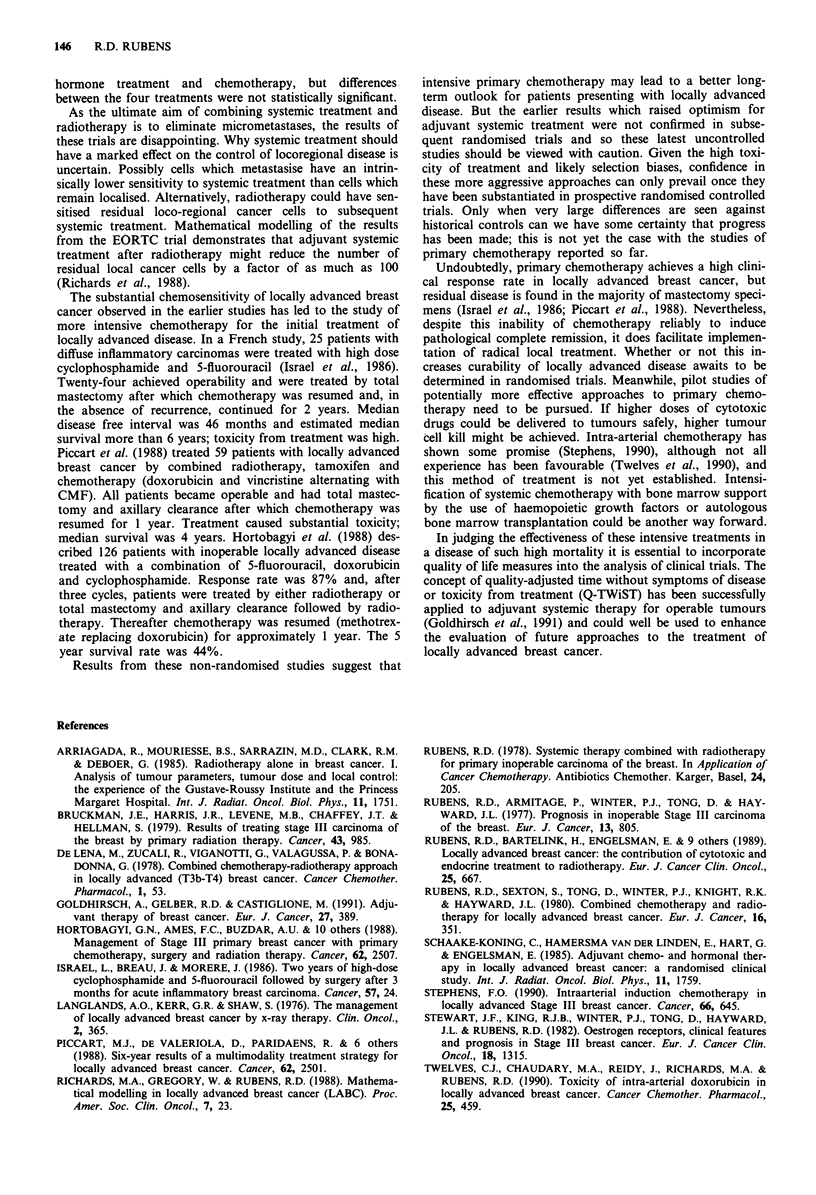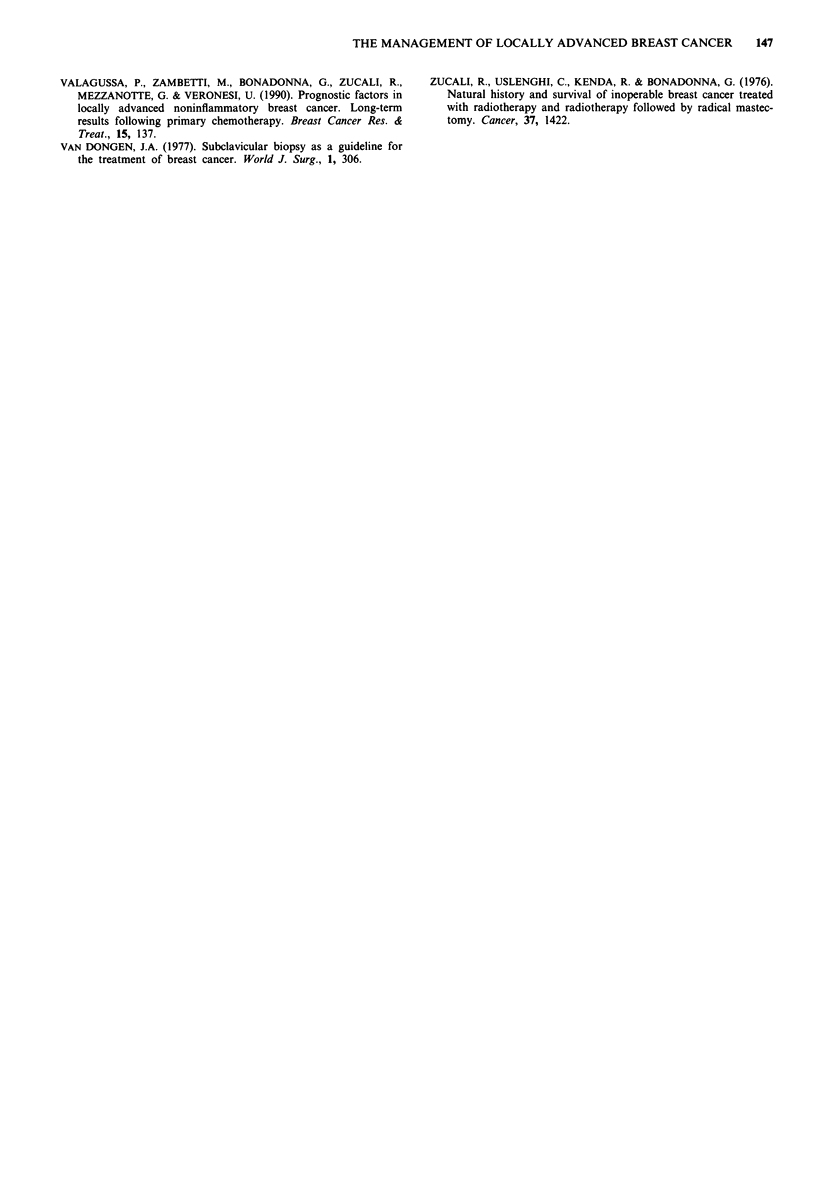# The management of locally advanced breast cancer.

**DOI:** 10.1038/bjc.1992.31

**Published:** 1992-02

**Authors:** R. D. Rubens


Br. J. Cancer (1992), 65, 145–147 © Macmillan Press Ltd., 1992

GUEST EDITORIAL

### The management of locally advanced breast cancer

R.D. Rubens

*ICRF Department of Clinical Oncology, UMDS, Guy's Hospital, London SE1 9RT, UK.*

Locally advanced breast cancer carries a high mortality. In a literature survey covering nearly 2,000 patients, the survival at 5 years was only 20% (Rubens, 1978). This stage of the disease usually reflects delay in presentation, although occasionally it takes a rapidly fulminating or 'inflammatory' form. With increasing public awareness of breast cancer and its treatment, we may expect the incidence of locally advanced disease steadily to diminish. For the time being, it accounts for about 15% of new cases of breast cancer in the UK, estimated to affect some 3,500 patients each year, while in developing countries the majority of patients present at this stage.

Locally advanced disease is characterised by the presence of one or more of the following features: infiltration of overlying skin, satellite skin nodules, extensive peau d'orange, attachment to deep structures, tethering or fixation of axillary nodes, involvement of supraclavicular nodes. It corresponds approximately to stage III of the TNM classification which encompasses T3,4, any N, M0 or any T, N2,3, M0 tumours. This includes some operable tumours, namely T3a, N0,1, M0, which are in this category solely because of the large size of the primary tumour. The other categories in stage III, T3b,4, N0,1, M0 or T0-4, N2,3, M0, are generally considered inoperable and usually treated by radiotherapy. Inoperable stage III tumours have a significantly worse prognosis than operable disease (Stewart *et al.*, 1982).

Radiotherapy for primary locally advanced breast cancer is to the whole breast, chest wall and the ipsilateral axillary, supraclavicular, infraclavicular and internal mammary nodes. Dose and schedule may vary from centre to centre, but not the basic principles. For example, the breast and chest wall may be treated tangentially and the draining lymph nodes irradiated in continuity by adjacent semi-opposed fields. Doses of up to 50 Gy are used with boosts approaching 20 Gy to sites of initially palpable disease. Local control is directly related to the radiation dose and inversely to the tumour volume (Arriagada *et al.*, 1985). In operable breast cancer, involvement of the highest axillary lymph node, the 'apex node', is associated with a poor prognosis after mastectomy (van Dongen, 1977). In centres where apex node biopsy is performed in the staging of breast cancer, radiotherapy rather than surgery is the preferred treatment when the result is positive.

Several studies on prognostic factors in Stage III disease have been reported (Langlands *et al.*, 1976; Rubens *et al.*, 1977; Zucali *et al.*, 1976). For survival, favourable factors include a long duration of symptoms (>6 months) before presentation, deep fixation of the primary tumour, a good response to primary radiotherapy and the rendering of initially inoperable disease suitable for mastectomy. Unfavourable factors are the early postmenopausal years (1–5 years since last menstrual period), a short duration of symptoms before presentation (<6 months), diffuse primary tumours and inflammatory carcinoma. In inoperable locally advanced disease, the size of the primary tumour and the presence of skin involvement appear to be neutral factors for survival, but have implications for local control. Using these covariates in a multivariate analysis and weighting them appropriately, it is possible to define groups of patients with significantly differing prognoses (Rubens *et al.*, 1977). This emphasises the importance of considering these variables in the analysis of clinical trials in locally advanced breast cancer.

In Stage III disease, the high incidence of subsequent distant metastases, reaching almost 70% in one series (Rubens *et al.*, 1977), accounts for the poor survival of patients treated by radiotherapy alone. This has led to the study of systemic treatment for its primary management.

Two early studies showed that chemotherapy with doxorubicin and vincristine before radiotherapy for locally advanced breast tumours gave a response frequency of about 70% and historical comparisons suggested that survival might be improved (De Lena *et al.*, 1978; Rubens *et al.*, 1980). In Milan, this work was developed further in a randomised comparison of radiotherapy and radical mastectomy after primary chemotherapy (Valagussa *et al.*, 1990). There was no difference in either time to progressive disease or survival. But both groups received further chemotherapy after local treatment and this, compared to historical experience, was observed significantly to improve both end-points. Others also reported that adjuvant chemotherapy after primary radiotherapy resulted in significantly better local control and survival compared to radiotherapy alone in matched historical controls (Bruckman *et al.*, 1979).

Systemic treatment following radiotherapy has now been tested in two randomised controlled clinical trials. The first was a three arm trial in which 118 patients were randomised to either radiotherapy alone, radiotherapy followed by 12 courses of cyclophosphamide, methotrexate and 5-fluorouracil (CMF), or courses of doxorubicin and vincristine alternating with CMF before and after radiotherapy (Schaake-Koning *et al.*, 1985). Local control and survival were not different in the three treatment arms. The second trial was conducted by the EORTC Breast Cancer Co-operative Group (Rubens *et al.*, 1989). Its aim was to test the independent and combined contributions of cytotoxic chemotherapy and endocrine therapy to radiotherapy in the primary treatment of locally advanced disease. In a factorial design, radiotherapy was the initial treatment for all patients after which they were randomly allocated to either radiotherapy alone, radiotherapy + endocrine therapy, radiotherapy + chemotherapy, or radiotherapy + endocrine therapy + chemotherapy. Endocrine treatment depended on menstrual status; premenopausal patients received ovarian irradiation and prednisolone whilst postmenopausal patients were given tamoxifen 10 mg bd for 5 years. Those randomised to receive chemotherapy had 12 cycles of CMF. In 363 evaluable patients, time to first progression was delayed significantly by either endocrine treatment or chemotherapy. Unexpectedly, the effect was due almost entirely to a delay in time to locoregional progression, for which the result was highly significant, rather than time to distant metastases. For survival, there was a trend in favour of the combination of

Received 30 August 1991.

hormone treatment and chemotherapy, but differences between the four treatments were not statistically significant.

As the ultimate aim of combining systemic treatment and radiotherapy is to eliminate micrometastases, the results of these trials are disappointing. Why systemic treatment should have a marked effect on the control of locoregional disease is uncertain. Possibly cells which metastasise have an intrinsically lower sensitivity to systemic treatment than cells which remain localised. Alternatively, radiotherapy could have sensitised residual loco-regional cancer cells to subsequent systemic treatment. Mathematical modelling of the results from the EORTC trial demonstrates that adjuvant systemic treatment after radiotherapy might reduce the number of residual local cancer cells by a factor of as much as 100 (Richards *et al.*, 1988).

The substantial chemosensitivity of locally advanced breast cancer observed in the earlier studies has led to the study of more intensive chemotherapy for the initial treatment of locally advanced disease. In a French study, 25 patients with diffuse inflammatory carcinomas were treated with high dose cyclophosphamide and 5-fluorouracil (Israel *et al.*, 1986). Twenty-four achieved operability and were treated by total mastectomy after which chemotherapy was resumed and, in the absence of recurrence, continued for 2 years. Median disease free interval was 46 months and estimated median survival more than 6 years; toxicity from treatment was high. Piccart *et al.* (1988) treated 59 patients with locally advanced breast cancer by combined radiotherapy, tamoxifen and chemotherapy (doxorubicin and vincristine alternating with CMF). All patients became operable and had total mastectomy and axillary clearance after which chemotherapy was resumed for 1 year. Treatment caused substantial toxicity; median survival was 4 years. Hortobagyi *et al.* (1988) described 126 patients with inoperable locally advanced disease treated with a combination of 5-fluorouracil, doxorubicin and cyclophosphamide. Response rate was 87% and, after three cycles, patients were treated by either radiotherapy or total mastectomy and axillary clearance followed by radiotherapy. Thereafter chemotherapy was resumed (methotrexate replacing doxorubicin) for approximately 1 year. The 5 year survival rate was 44%.

Results from these non-randomised studies suggest that intensive primary chemotherapy may lead to a better long-term outlook for patients presenting with locally advanced disease. But the earlier results which raised optimism for adjuvant systemic treatment were not confirmed in subsequent randomised trials and so these latest uncontrolled studies should be viewed with caution. Given the high toxicity of treatment and likely selection biases, confidence in these more aggressive approaches can only prevail once they have been substantiated in prospective randomised controlled trials. Only when very large differences are seen against historical controls can we have some certainty that progress has been made; this is not yet the case with the studies of primary chemotherapy reported so far.

Undoubtedly, primary chemotherapy achieves a high clinical response rate in locally advanced breast cancer, but residual disease is found in the majority of mastectomy specimens (Israel *et al.*, 1986; Piccart *et al.*, 1988). Nevertheless, despite this inability of chemotherapy reliably to induce pathological complete remission, it does facilitate implementation of radical local treatment. Whether or not this increases curability of locally advanced disease awaits to be determined in randomised trials. Meanwhile, pilot studies of potentially more effective approaches to primary chemotherapy need to be pursued. If higher doses of cytotoxic drugs could be delivered to tumours safely, higher tumour cell kill might be achieved. Intra-arterial chemotherapy has shown some promise (Stephens, 1990), although not all experience has been favourable (Twelves *et al.*, 1990), and this method of treatment is not yet established. Intensification of systemic chemotherapy with bone marrow support by the use of haemopoietic growth factors or autologous bone marrow transplantation could be another way forward.

In judging the effectiveness of these intensive treatments in a disease of such high mortality it is essential to incorporate quality of life measures into the analysis of clinical trials. The concept of quality-adjusted time without symptoms of disease or toxicity from treatment (Q-TWiST) has been successfully applied to adjuvant systemic therapy for operable tumours (Goldhirsch *et al.*, 1991) and could well be used to enhance the evaluation of future approaches to the treatment of locally advanced breast cancer.

### References

ARRIAGADA, R., MOURIESSE, B.S., SARRAZIN, M.D., CLARK, R.M. & DEBOER, G. (1985). Radiotherapy alone in breast cancer. I. Analysis of tumour parameters, tumour dose and local control: the experience of the Gustave-Roussy Institute and the Princess Margaret Hospital. *Int. J. Radiat. Oncol. Biol. Phys.*, **11**, 1751.

BRUCKMAN, J.E., HARRIS, J.R., LEVENE, M.B., CHAFFEY, J.T. & HELLMAN, S. (1979). Results of treating stage III carcinoma of the breast by primary radiation therapy. *Cancer*, **43**, 985.

DE LENA, M., ZUCALI, R., VIGANOTTI, G., VALAGUSSA, P. & BONADONNA, G. (1978). Combined chemotherapy-radiotherapy approach in locally advanced (T3b-T4) breast cancer. *Cancer Chemother. Pharmacol.*, **1**, 53.

GOLDHIRSCH, A., GELBER, R.D. & CASTIGLIONE, M. (1991). Adjuvant therapy of breast cancer. *Eur. J. Cancer*, **27**, 389.

HORTOBAGYI, G.N., AMES, F.C., BUZDAR, A.U. & 10 others (1988). Management of Stage III primary breast cancer with primary chemotherapy, surgery and radiation therapy. *Cancer*, **62**, 2507.

ISRAEL, L., BREAU, J. & MORERE, J. (1986). Two years of high-dose cyclophosphamide and 5-fluorouracil followed by surgery after 3 months for acute inflammatory breast carcinoma. *Cancer*, **57**, 24.

LANGLANDS, A.O., KERR, G.R. & SHAW, S. (1976). The management of locally advanced breast cancer by x-ray therapy. *Clin. Oncol.*, **2**, 365.

PICCART, M.J., DE VALERIOLA, D., PARIDAENS, R. & 6 others (1988). Six-year results of a multimodality treatment strategy for locally advanced breast cancer. *Cancer*, **62**, 2501.

RICHARDS, M.A., GREGORY, W. & RUBENS, R.D. (1988). Mathematical modelling in locally advanced breast cancer (LABC). *Proc. Amer. Soc. Clin. Oncol.*, **7**, 23.

RUBENS, R.D. (1978). Systemic therapy combined with radiotherapy for primary inoperable carcinoma of the breast. In *Application of Cancer Chemotherapy*. Antibiotics Chemother. Karger, Basel, **24**, 205.

RUBENS, R.D., ARMITAGE, P., WINTER, P.J., TONG, D. & HAYWARD, J.L. (1977). Prognosis in inoperable Stage III carcinoma of the breast. *Eur. J. Cancer*, **13**, 805.

RUBENS, R.D., BARTELINK, H., ENGELSMAN, E. & 9 others (1989). Locally advanced breast cancer: the contribution of cytotoxic and endocrine treatment to radiotherapy. *Eur. J. Cancer Clin. Oncol.*, **25**, 667.

RUBENS, R.D., SEXTON, S., TONG, D., WINTER, P.J., KNIGHT, R.K. & HAYWARD, J.L. (1980). Combined chemotherapy and radiotherapy for locally advanced breast cancer. *Eur. J. Cancer*, **16**, 351.

SCHAAKE-KONING, C., HAMERSMA VAN DER LINDEN, E., HART, G. & ENGELSMAN, E. (1985). Adjuvant chemo- and hormonal therapy in locally advanced breast cancer: a randomised clinical study. *Int. J. Radiat. Oncol. Biol. Phys.*, **11**, 1759.

STEPHENS, F.O. (1990). Intraarterial induction chemotherapy in locally advanced Stage III breast cancer. *Cancer*, **66**, 645.

STEWART, J.F., KING, R.J.B., WINTER, P.J., TONG, D., HAYWARD, J.L. & RUBENS, R.D. (1982). Oestrogen receptors, clinical features and prognosis in Stage III breast cancer. *Eur. J. Cancer Clin. Oncol.*, **18**, 1315.

TWELVES, C.J., CHAUDARY, M.A., REIDY, J., RICHARDS, M.A. & RUBENS, R.D. (1990). Toxicity of intra-arterial doxorubicin in locally advanced breast cancer. *Cancer Chemother. Pharmacol.*, **25**, 459.

VALAGUSSA, P., ZAMBETTI, M., BONADONNA, G., ZUCALI, R., MEZZANOTTE, G. & VERONESI, U. (1990). Prognostic factors in locally advanced noninflammatory breast cancer. Long-term results following primary chemotherapy. *Breast Cancer Res. & Treat.*, **15**, 137.

VAN DONGEN, J.A. (1977). Subclavicular biopsy as a guideline for the treatment of breast cancer. *World J. Surg.*, **1**, 306.

ZUCALI, R., USLENGHI, C., KENDA, R. & BONADONNA, G. (1976). Natural history and survival of inoperable breast cancer treated with radiotherapy and radiotherapy followed by radical mastectomy. *Cancer*, **37**, 1422.